# Professor Paine’s Introductory Lecture

**Published:** 1845-04

**Authors:** 


PROFESSOR PAINE'S INTRODUCTORY LECTURE.

The war still rages between the vitalists and the chemists, but, it would appear, must soon come to an end, for nearly all the combatants are on one side. Now and then a physiologist may be found hardy enough to deny chemistry all connexion with the phenomena of life—to assert that "the union between the schools of vitalism and chemistry, would be as unnatural as human brains in a block of granite;" but such individuals are rare, and the number is not increasing. Professor Paine, of New York, who devoted his late introductory lecture to the demolition of Liebig's theory of digestion, is one of the few who hold these doctrines, and it is in his lecture that we find the words just quoted. His effort in this lecture is to convict Liebig of self-contradiction, holding with the reviewer of his "Commentaries," that "a writer who can so contradict himself, scarcely needs to be exposed." We must say that we do not consider his effort a successful one. However just the charge of contradicting himself may have been, as made by Carpenter against Paine, and about this we express no opinion, it is not applicable to Liebig. True, that eminent chemist occasionally finds it necessary in the progressive developments of science to change his views, and to give up certain positions which he had previously assumed; but the contradictions of which this lecture professes to make such a display, we are compelled to believe, exist no where but in the imagination of the critic. Not that the language of Liebig is always unambiguous, and may not in places be so interpreted as to make out inconsistencies; but taken in the sense meant to be conveyed by the author, it teaches a doctrine every where consistent with itself. We have looked in vain through his writings for the passages exhibiting that "medley of contradictions" which Dr. Paine strives to make appear. Those which he arrays in parallel columns are not contradictory if we give to the words the meaning attached to them by Liebig; and it is in this spirit that every author must be read if we would do him justice, or even understand him.

This lecture is not in good taste. Its tone is extravagant, and sometimes almost acrimonious. The author in the heat of controversy has been betrayed into the use of language which his own deliberate judgment would condemn. In his cooler moments, we are persuaded, he would not charge the chemists with a wish to deceive: with affecting to believe in the existence of a vital principle, but "*in reality*" discarding all but "pure chemistry" from their system of physiology—with throwing out the doctrine of a vital principle "as springes to catch wood-cocks," or as "a tub to the whale." Neither would he be guilty of the offence of applying to the doctrines of the chemists the term "stultifying." He would feel that there was no force in arguments consisting of abusive epithets, but that in the use of such weapons as these a philosopher would find his match in a very stupid antagonist.

Animal chemistry has nothing to fear from such attacks as those of which the one made upon it in this lecture is a sample. They may mistify or mislead the minds of a few medical students, but that is all the mischief they will do. Y.

